# Post-Treatment Displacement of Facial Soft Tissue Fillers—A Retrospective Ultrasound-based Investigation of 382 Zygomatic Regions

**DOI:** 10.1097/DSS.0000000000004257

**Published:** 2024-06-04

**Authors:** Leonie Schelke, Nicola Lowrey, Ali Mojallal, MJ Rowland-Warmann, Ximena Wortsman, Rosa M. Sigrist, Peter J. Velthuis, Sebastian Cotofana

**Affiliations:** * Department of Dermatology, Erasmus University Medical Center, Rotterdam, The Netherlands;; † Department of Plastic, Reconstructive, and Aesthetic Surgery, N2 Aesthetics, Manhattan Beach, CA;; ‡ Department of Plastic, Reconstructive, and Aesthetic Surgery, Hospices Civils de Lyon, Lyon, France;; § Department of Plastic, Reconstructive, and Aesthetic Surgery, Université Claude Bernard, Lyon, France;; ║ Department of Plastic, Reconstructive, and Aesthetic Surgery, Smileworks Liverpool, Liverpool, UK;; ¶ Department of Plastic, Reconstructive, and Aesthetic Surgery, Institute for Diagnostic Imaging of the Skin and Soft Tissues, Santiago, Chile;; ** Department of Dermatology, Universidad de Chile, Santiago, Chile;; †† Department of Radiology, Faculty of Medicine, University of São Paulo, São Paulo, Brazil;; ‡‡ Centre for Cutaneous Research, Blizard Institute, Queen Mary University of London, London, UK;; §§ Department of Plastic and Reconstructive Surgery, Guangdong Second Provincial General Hospital, Guangzhou, Guangdong Province, China

## Abstract

**BACKGROUND:**

Clinical and ultrasound experience has revealed that after soft tissue injections of the lateral cheek, the filler may displace from the zygoma to the caudal temporal area.

**OBJECTIVE:**

To obtain more data to provide insight into product distribution when soft tissue fillers are injected in the zygomatic region.

**METHODS:**

Two hundred patients were examined with facial ultrasound imaging of the zygomatic and temporal region. Inclusion criteria were simply a positive response on the screening questionnaire as to whether or not they had filler injections placed in their lateral cheek. Control injections were also performed to the zygomatic regions of a body donor and in 10 patients ultrasound-guided.

**RESULTS:**

A correlation was found between the layers in which filler was detected on the zygoma and where it was ultimately found in the temples. Four different redistribution patterns were observed: (1) migration of filler within the superficial muscular aponeurotic system (SMAS) on the zygoma into the superficial temporal fascia. Migration of filler from the lateral suborbicularis oculi fat to (2) the deep interfacial plane of the temple or (3) to the superficial temporal fat pad; (4) migration from the supraperiosteal layer of the zygoma to the superficial temporal fat pad. Body donor and patients: filler deposits injected on the zygoma were witnessed to shift during injection into the caudal part of the temple.

**CONCLUSION:**

Soft tissue filler aliquots may be redistributed into the temples after injections of the lateral side of the zygomatic arch. The displacement follows a distinct pattern depending on the initial layer of injection.

## Abstract 2

Supplemental Digital Content is Available in the Text.

Soft tissue filler injections are widely accepted for the use of minimally invasive aesthetic procedures. They are frequently used to reposition facial soft tissues,^1^ for volumization,^2^ to restructure age-related facial features like the nasolabial fold,^3–5^ and to change the facial shape when addressing the jawline and the mandibular angle.^3,4^

The most frequently targeted facial regions are the lips, nasolabial folds, and the midface/cheeks. The midface region can be further divided into the medial and lateral cheek, which has the power to impact the entire facial shape, differentiate between feminine or masculine facial appearance, and can co-influence the nasolabial fold when targeted appropriately with soft tissue filler injections.^2,6–8^

Anatomically, the medial and the lateral cheeks are separated by the line of ligaments. Behind the line of ligaments, in the lateral cheek and extending into the temple region, the subdermal fascial layers are arranged in parallel orientation to the skin surface. Whereas the layers are arranged differently in front of the line of ligaments in the medial cheek.^9^

The medial cheek, also known as the malar region, lies in front of the line of ligaments. Here, seven layers can be identified (skin, superficial fatty layer, superficial musculo-aponeurosis system, suborbicularis oculi fat, mid facial extension of the superficial laminae of the deep temporal fascia, preperiosteal fat located within the prezygomatic space, and periosteum).^9–11^ Lateral to this region along the zygomatic arch, only 5 layers can be identified (skin, superficial fatty layer, superficial musculoskeletal-aponeurotic system, deep supraperiosteal fat, and periosteum).^12^ Additionally, the lateral cheeks have shown to have direct connections with the temples via the temporal tunnel and the fascial continuation of the 13 layers of the temple.^13,14^

Recent publications have indicated that the distribution of soft tissue filler material is dependent on many factors. These factors will ultimately influence the final product location. It has been found that of those influencing factors, the most relevant are needle versus cannula use,^15,16^ gauge or external diameter measurement of the needle or cannula,^17^ rheologic properties of the product,^18,19^ injection angle,^17^ and variables such as injection speed and needle/cannula movement.^20^

Clinical and ultrasound experience has additionally revealed that after injections of the lateral cheek region, the administered product can be identified within the temple or inside the more superficial fascial layers of the zygomatic region. This has been visualized even when the initial soft tissue filler injection was deposited with the needle or cannula in contact with the bone in the supraperiosteal plane.

Before now, only clinical or anecdotal evidence was available for these claims of filler redistribution. Therefore, the present study was designed to provide more robust and reliable data. True analysis of these clinical cases can provide insight into product distribution and into the precision of soft tissue filler injections for the zygomatic region.

## Material and Methods

### Study Setup

Two hundred volunteers were recruited for this study. Many were attendees at international conferences (IMCAS 2023) Paris (L.S., N.L., M.J.R., R.S., X.W.), Scale 2023, Nashville (L.S., N.L.), Season 2023 London (L.S., P.V.), Facial Ultrasound Courses (L.S., N.L.). The remaining were patients at an outpatient clinic for filler complications, Rotterdam (L.S.), and in private medical practices in Los Angeles (N.L.), and Amsterdam (L.S.). Patients and the participants from the conferences were approached and asked for their willingness to participate in this study. Upon agreement, demographic and aesthetic medical data were collected. Then, a facial ultrasound scan of their zygomatic and temporal region was conducted.

Inclusion criteria for the final study analysis were volunteers that answered positively to the question of whether they had previous filler injections to their lateral cheeks. All volunteers included in this study provided written informed consent for the use of their demographic, medical, and imaging data for the purposes of this study. Ethics committee approval to gather data concerning soft tissue filler complications was obtained (MEC-2016-0660).

### Body Donor Injections

For reference, 2 control injections were performed to the zygomatic region of a body donor (Laboratoire d'anatomie, Rockefeller Medical school, Lyon, France) using a high G-prime product that was colored with methylene blue for visualization purposes (Vivacy, France). For the injection procedure, a 27G needle was used, positioned to touch the bone and the product applied while in constant contact with the bone, under low injection pressure. After injection, anatomical dissections were conducted to identify the plane and extent of product distribution.

### Ultrasound Guided Filler Injections

For additional reference, 10 patients were injected under ultrasound guidance with hyaluronic acid filler (Galderma Restylane Volyme and Allergan Juvederm Voluma). The product was injected supraperiostally on the zygoma. In six patients, a 25G needle was used with an injection angle of 30°. In the other four patients, a 27G needle was used, with an injection angle of 90°, perpendicular to the bone. Three boluses of 0.1 mL were injected on the lateral zygomatic bone of each patient. The distribution of the filler during injections was able to be visualized and identified on the ultrasound images.

### Ultrasound Imaging

All ultrasound examiners were experienced in facial ultrasound anatomy and filler recognition. With a high frequency MHz probe (20 MHz linear probe GE Healthcare Venue Go and 18 MHz linear probe Philips Affinity 70), the zygoma and temple areas on both sides of the face were examined. A total of 400 scans were completed.

At the onset of the exam, the probe was placed in a horizontal position on the zygomatic arch. When the filler was identified, the probe was rotated to an oblique-vertical position on the caudal temple area to search for an extension of filler substance into the temple. Images were taken, stored, and later assessed independently by two physicians experienced in reading US images (L.S., P.V.).

The filler substance location was determined to be

(1) On the zygoma in (1) the superficial fatty layer, (2) in the fibrous layers of the fascia/superficial muscular aponeurotic system (SMAS), (3) in the sub-SMAS (deep fat/supraperiosteal plane), (4) in the muscle, and (5) “other layer.”
(2) In the temples in (1) the subdermal fatty layer, (2) the superficial temporal fascia, (3) the interfascial plane, (4) the deep interfascial plane, (5) the superficial temporal fat pad, (6) the temporalis muscle, (7) the deep temporal fat pad, (8) the periosteum, and (9) “other layer.”

### Statistical Analysis

The data were processed with IBM-SPSS26 (IBM Corp., 2017, Armonk, NY) to calculate the basic statistical parameters (percentages, frequencies).

## Results

### Sample Description

**Figure 1.**
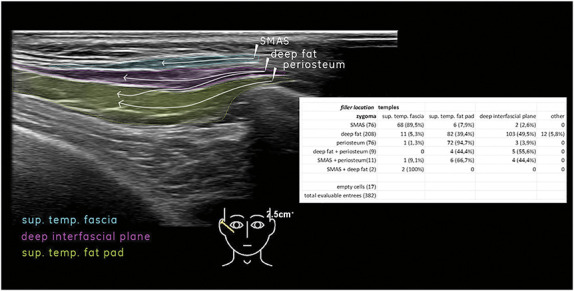
Filler migration patterns from locations on zygoma to locations in temples.

A total of 200 volunteers (9 men, 191 women; with a mean age of 47.6 years [10.5]) were included in this retrospective investigation. Of the 400 zygoma regions examined, data quality was considered insufficient in 17 cases. For example, in one case, only a unilateral product deposition was identified during the ultrasound scanning. Hence, data from 382 zygomatic regions were ultimately included in this retrospective analysis. Injected substances are given in Figure 1.

### Clinical Findings

Given the nature of this investigation being a retrospective analysis of previously injected cases, participants inconsistently recalled the accurate date of their original aesthetic injections. It is important to note that the medical and aesthetic history of the 200 volunteers for this study were not clinically reviewed based on their patient charts. Participants were largely recruited at professional conferences, and their history was collected during verbal interviews. Therefore, the detailed interval between the date of injection and the date of ultrasound investigation cannot be provided here.

### Adverse Events

Patients reported no serious adverse events that would have suggested a vascular compromise during the initial injection procedure. However, mild complaints such as a feeling of pressure (*N* = 2 [1%]), edema (*N* = 7 [3.5%]), dissatisfaction with appearance because of overfilling (*N* = 17 [8.5%]), or telangiectasis (*N* = 4 [2%]) were described.

### Ultrasound Findings

On ultrasound imaging, hyaluronic acid (HA) filler was seen as anechoic to hypoechoic well-defined oval-shaped deposit(s), sometimes with posterior enhancement. This is in contrast to calcium hydroxyapatite and poly-l-lactic acid, which presents as ill-defined heterogenous hyperechoic mass-like deposits on a hypoechoic background. Polyalkylmimide hydrogel was a mass-like hypoechoic deposit with or without internal echoes. Polymethyl-methacrylate exhibits as a hyperechoic band-like structure with mini comet tail artifact and sometimes posterior shadowing.^21^

**Figure 2.**
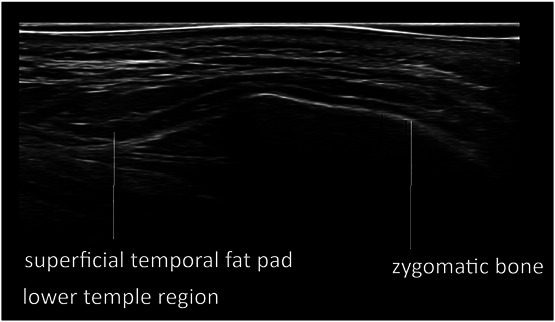
Ultrasound (grayscale, transverse view) of the zygomatic and temple area (Philips affinity 70 18 MHz).

In general, fat layers are seen as lobulated hypoechoic tissues separated by hyperechoic linear (fibrous) septa. The SMAS and fascia are characterized by hyperechoic linear sheets of variable thickness with a clear fibrillation pattern. Facial dynamic muscles such as the orbicularis oculi and the temporalis are hypoechoic band-like structures. A continuity in planes is observed between the zygomatic arch and the lower temple region (Figure 2). Deep fat often has the same echogenicity as hyaluronic acid and some individuals may have large fat lobules, which can make differentiating the 2 confusing. To distinguish between HA deposits and fat lobules, they were scanned in different axes. Comparing the images from the individuals with and those without fillers in this manner will distinguish well-defined (in all axes) oval-shaped HA deposits that can be differentiated from fat lobules.

A clear correlation was found between the layers in which filler was detected on the zygoma and subsequently in the temple (Figure 1). With ultrasound imaging, filler material was often found at the zygomatic arch, where it was originally injected. Subsequently, a redistribution of the filler material into the caudal temporal area was seen in all cases except four. On inspection, the redistributed filler material was only visualized in the lower or caudal part of the temporal area.

**Figure 3.**
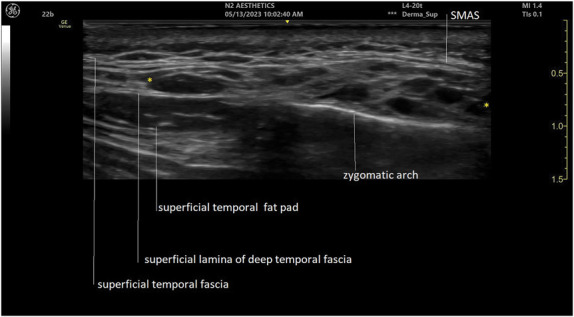
Ultrasound (grayscale, transverse view) of the zygomatic and temple area showing filler material injected in the deep fat of the lateral SOOF shifts into the deep interfascial plane of the temple. Hyaluronic acid deposits in between yellow markers (GE venue Go 20 MHz). SOOF, suborbicularis oculi fat.

**Figure 4.**
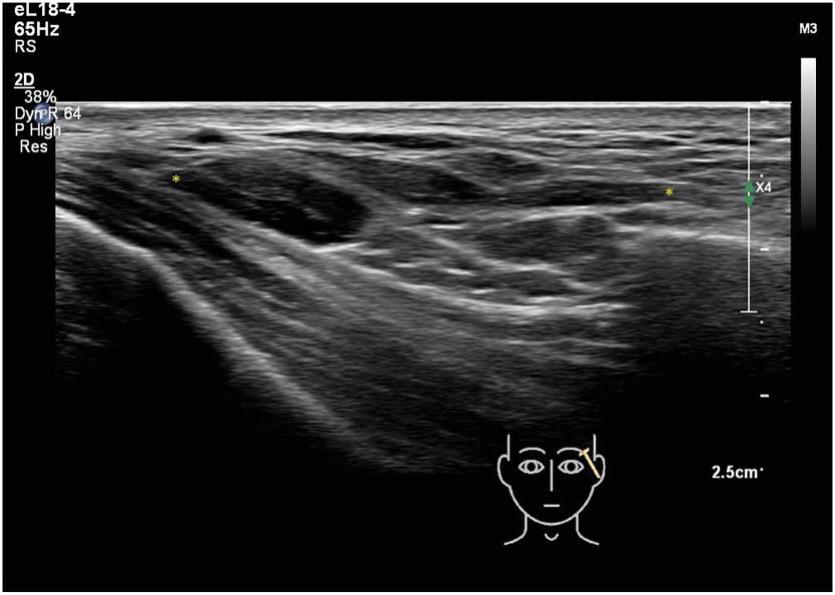
Ultrasound (grayscale, oblique view) of the temple area depicting filler material injected in the deep fat of the lateral SOOF shifts into the superficial temporal fat pad of the temple. Hyaluronic acid deposits in between yellow markers (Philips affinity 70 18 MHz). SOOF, suborbicularis oculi fat.

**Figure 5.**
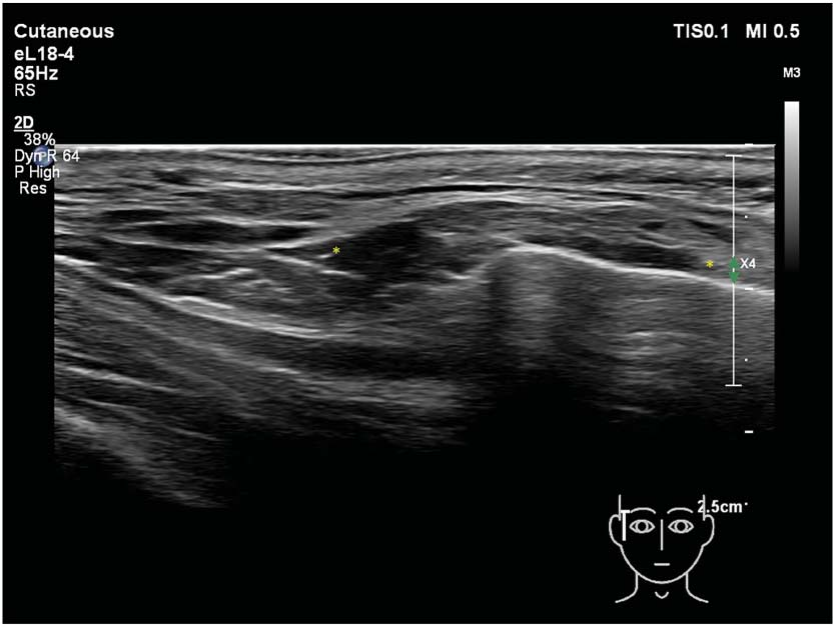
Ultrasound (grayscale, longitudinal view) of the temple and zygomatic area showing filler material injected at the periosteum of the zygomatic bone shifts into the superficial temporal fat pad. Hyaluronic acid filler in between yellow markers (Philips affinity 70 18 MHz).

**Figure 6.**
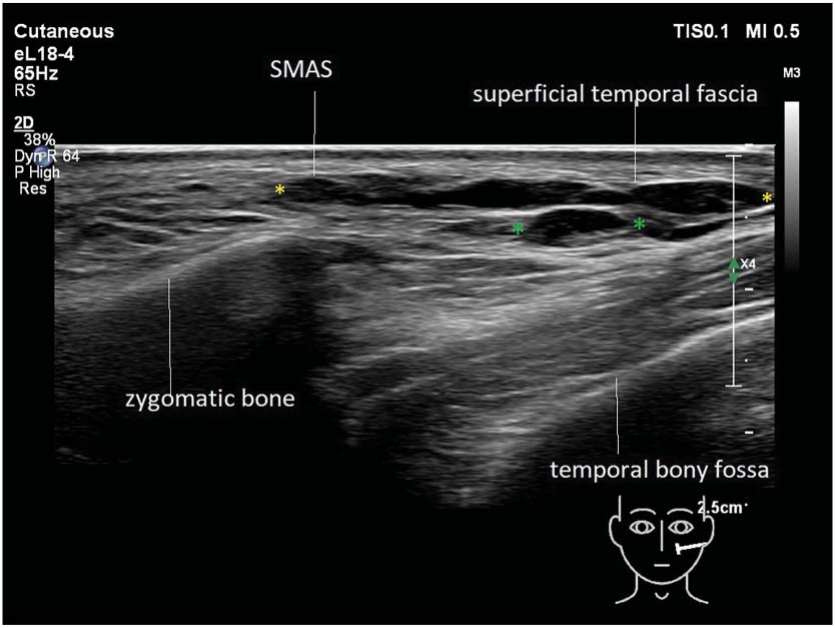
Ultrasound (grayscale, oblique view) of the temple area depicting filler material injected into the SMAS distributed into the superficial temporal fascia (yellow markers) and some filler material in the deep interfascial plane (green markers [Philips affinity 70 18 MHz]).

Four different primary patterns of potential redistribution were suspected (Figures 3–6). In many cases, filler material redistributed to more than one tissue layer. (1) Filler initially placed between the layers of the fascia/SMAS on the zygoma displaced to the superficial temporal fascia (89.5%). Filler placed in the lateral suborbicularis oculi fat (SOOF) displaced to either (2) the deep interfascial plane of the temple (49.5%) or (3) the superficial temporal fat pad (39.4%). (4) Filler initially placed on the supraperiosteal level of the zygoma displaced to the superficial temporal fat pad (94.7%). On the whole, filler material originally placed to the zygoma was evidenced in the superficial temporal fat pad (44.5%), in the deep interfascial plane of the temple (30.6%), in the superficial temporal fascia (21.7%), or in other areas (3.1%).

### Body Donor Findings

**Figure 7.**
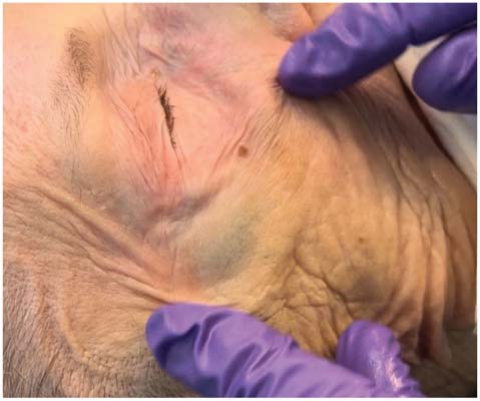
Fresh frozen body donor head (Laboratoire d'anatomie, Rockefeller Medical School, Lyon, France). Colored filler aliquots shifted during injection on the zygomatic arch into the temporal area.

**Figure 8.**
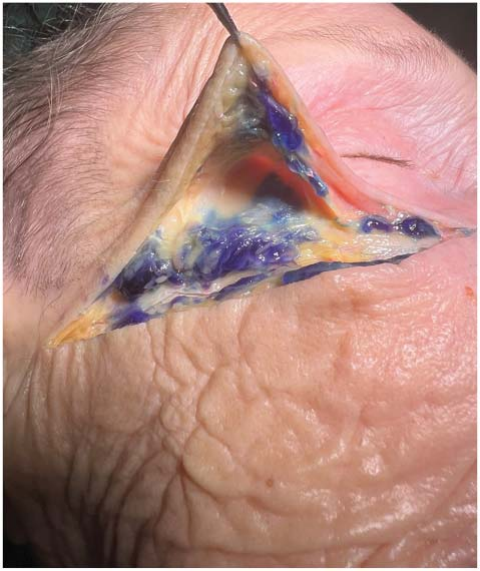
Fresh frozen body donor head (Laboratoire d'anatomie, Rockefeller Medical School, Lyon, France). Colored filler aliquots redistributed during injection on the zygomatic arch into the caudal part of the superficial temporal fat pad (black arrow).

After the supraperiosteal injections of HA onto the donor's zygoma, direct visualization confirmed that one filler deposit (0.2 mL of HA) remained at the injection site, while the second injection (0.3 mL of HA) displaced during injection into the caudal part of the superficial fat compartment of the temple (Figures 7 and 8)

### Patients Findings

After ultrasound-guided injections of HA supraperiosteal on the zygoma, visualization with ultrasound confirmed that in all patients the most lateral filler deposit (0.1 mL of HA), due to backflow of filler material, displaced during injection into the caudal part of the temples (see **Supplemental Digital Content 1**, Video 1, http://links.lww.com/DSS/B458).

## Discussion

The subdermal fascial layers are arranged in parallel orientation to the skin surface both in the temple and in the lateral midface. This results in a connection between the temporal and mid facial structures, which can influence the aesthetic outcome. The skin and the superficial (subdermal) fatty layer are continuous with each other and do not have a different nomenclature to distinguish the two anatomic regions. The next layer, the superficial temporal fascia (= layer 3), is continuous with the SMAS of the midface and with the orbicularis oculi muscle in the periorbital region. Deep to the superficial temporal fascia, the deep fat of the temple can be identified. This fat is located in the interfacial plane (= layer 4) and is continuous with the deep fat of the lateral midface and with the SOOF of the malar region. The interfascial plane contains the motor branches of the facial nerve and is the plane located between the superficial temporal fascia and the superficial lamina of the deep temporal fascia (= layer 6).^2,22–24^ The latter is continuous into the midface as the midfacial extension of the superficial lamina of the deep temporal fascia and separates the SOOF from the preperiosteal fat located within the prezygomatic space. The superficial temporal fat pad (also termed the intermediate fat pad of the temple; layer 7) is located between the superficial (= layer 6) and the deep lamina (layer = 8) of the deep temporal fascia and extends and envelopes the zygomatic arch from its cranial aspect. Historically, it was thought not to extend into the lateral midface.

This observational study reveals that soft tissue filler material injected alongside the zygomatic arch invariably redistributes to the lower temple region after distinct migration pathways. Primarily, the product ends up in the superficial temporal fat pad, but also in the deep interfascial plane, and in the superficial temporal fascia. This visualized finding was unexpected, in particular, since the periosteum of the zygomatic bone is supposed to be continuous with the deep temporal fascia. With filler injections in that area, it is customary to have direct bone contact with the needle tip. Therefore, the only expected route for redistribution was to the interfascial plane. Considering many variations were observed, a correlation was established between the primary layer of filler placement to the zygoma, and the layer where filler was found in the temples. Thus, a clear anatomical pathway of filler redistribution from zygoma to temples may be assumed.

The intended result of filler material delivered to the zygomatic arch is a small oval-shaped aliquot on the periosteum. However, previous studies have shown that filler material is frequently not found in its intended location, as backflow of filler often occurs.^15,25^ For injections to reach the zygomatic bone, a needle or cannula must penetrate the fibrous SMAS. In doing this, a tract is created allowing filler material placed on the periosteum to flow backward into the more superficial layers. To clarify, it is expected that when injecting filler sub-SMAS, some filler material will eventually be found between the fibrous layers of the SMAS due to backflow.^26^ Alternatively, by aiming to inject on the periosteum, the tip of the needle is typically touching the bone, but in some cases, the needle may not have perforated the SMAS layers, instead the needle tip may only be pinning the SMAS down. Filler product is subsequently injected into one of the layers of the SMAS instead of placing it periosteally as intended.

Apart from the initial injection technique, several other factors will play a role in filler redistribution. These include the amount of filler used and its physical properties. At the zygomatic arch, the limited space and the tightness of the SMAS will also be a factor. Instead of one bolus delivered onto the bone at the injection site, the backward flow of filler along the zygomatic bone in the direction of the temple is evident. This was observed in patients treated ultrasound-guided. With this, it can be assumed that redistribution of filler from the sub-SMAS region into the temples is not injector-related but instead related to the anatomy of the area. However, in other cases, filler material was also found to be redistributed from the SMAS into the superficial temporal fascia. Unlike the sub-SMAS injections, the authors consider this pathway to be injection-technique related.

Both patients and injectors are usually pleased with the outcome of filler injections on the zygoma. It is, therefore, questionable whether this study points to any matter of clinical relevance. Most volunteers in this study experienced no adverse events. Only a minority sought follow-up care because of complaints of pressure, pain, dilated veins, or dissatisfaction with the aesthetic outcome.

**Figure 9.**
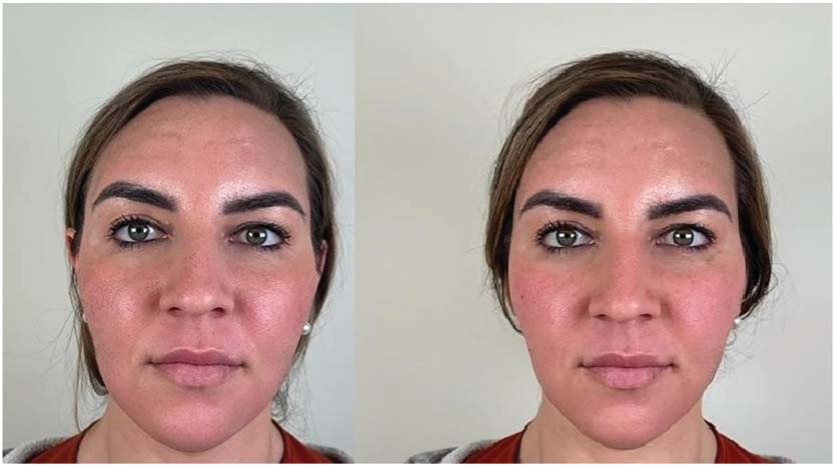
Left before and right side after dissolving HA deposits of the caudal temporal area. Note the more natural appearance when the face becomes slimmer. HA, hyaluronic acid.

As a clinical observation, inadvertent filling of the caudal temple may create a undesirable increase in the width of the midface, leading to increased concavity of the cranial part of the temples.^27^ This may become more evident after the filler in the caudal part of the temple is dissolved (Figure 9). When asked specifically, volunteers in this study often noticed and disliked this wider facial structure. Furthermore, and importantly, it should be taken into consideration that the main purpose for filler placement at the zygomatic arch is to lift and support the facial ligaments. Injections on the zygomatic bone will not provide the intended lifting or supportive effect when the material shifts into the temple area.

This study has not considered the type and amount of filler or the time lapse after injection. Also, the exact location of injections was not assessed. Therefore, it could not be noted whether the redistribution of the product is immediate or if it occurs over time. In the case of filler flow dynamics, ultrasound imaging seems superior to cadaver dissection studies. However, cadaver dissections of this area may be better suited to find the pathway(s) between the zygoma and the temple areas where the redistribution of filler can occur.

## Conclusion

Filler aliquots placed onto the lateral zygomatic arch may partly displace into the temples after injection. The redistribution of the product appears to follow a distinct pattern depending on the initial layer of injection.

## References

[1] Cotofana S Fratila AAM Schenck TL Redka-Swoboda W . The anatomy of the aging face: a review. Facial Plast Surg 2016;32:253–60. 27248022 10.1055/s-0036-1582234

[2] Cotofana S Gotkin RH Frank K Koban KC . The functional anatomy of the deep facial fat compartments: a detailed imaging-based investigation. Plast Reconstr Surg 2019;143:53–63. 30589776 10.1097/PRS.0000000000005080

[3] de Maio M. MD codes™: a methodological approach to facial aesthetic treatment with injectable hyaluronic acid fillers. Aesthet Plast Surg 2021;45:690–709. 10.1007/s00266-020-01762-7 PMC8012343 32445044

[4] de Maio M DeBoulle K Braz A Rohrich RJ . Facial assessment and injection guide for Botulinum toxin and injectable hyaluronic acid fillers: focus on the midface. Plast Reconstr Surg 2017;140:540e–550e. 10.1097/PRS.0000000000003716 28953721

[5] Swift A Liew S Weinkle S Garcia JK . The facial aging process from the “inside out”. Aesthet Surg J 2021;41:1107–19. 33325497 10.1093/asj/sjaa339 PMC8438644

[6] Duan J Zhao WR Luo CE Luo SK. Anatomical basis for malar augmentation injection with the zygomatic ligamentous system. Dermatol Surg 2022;48:1059–64. 35834641 10.1097/DSS.0000000000003537

[7] Li Z Xia Z Qiu Z Ting W . Studying dynamics of mid-face lifting during hyaluronic acid filler injection using ultrasound imaging. Aesthet Plast Surg 2023;47:2661–76. 10.1007/s00266-022-03221-x 36536094

[8] Cotofana S Schenck TL Trevidic P Sykes J . Midface: clinical anatomy and regional approaches with injectable fillers. Plast Reconstr Surg 2015;136:219S–234S. 26441102 10.1097/PRS.0000000000001837

[9] Casabona G Frank K Koban KC Freytag DL . Lifting vs volumizing-The difference in facial minimally invasive procedures when respecting the line of ligaments. J Cosmet Dermatol 2019;18:1237–43. 31402563 10.1111/jocd.13089

[10] Casabona G Bernardini FP Skippen B Rosamilia G . How to best utilize the line of ligaments and the surface volume coefficient in facial soft tissue filler injections. J Cosmet Dermatol 2020;19:303–11. 31840373 10.1111/jocd.13245

[11] Cong LY Duan J Luo CE Luo SK. Injectable filler technique for face lifting based on dissection of true facial ligaments. Aesthet Surg J 2021;41:NP1571–NP1583. 33300562 10.1093/asj/sjaa348

[12] Cotofana S Lachman N. Anatomy of the facial fat compartments and their relevance in aesthetic surgery. J Dtsch Dermatol Ges 2019;17:399–413. 10.1111/ddg.13737 30698919

[13] O'Brien JX Ashton MW Rozen WM Ross R . New perspectives on the surgical anatomy and nomenclature of the temporal region: literature review and dissection study. Plast Reconstr Surg 2013;131:510–22. 23165177 10.1097/PRS.0b013e31827c6ed6

[14] Lee HJ Kim HM Ahn HS Lee JH . Novel clinical anatomical consideration of the superficial and deep layers of the deep temporal fascia. Plast Reconstr Surg 2024;153:591–9. 37010473 10.1097/PRS.0000000000010507

[15] van Loghem JAJ Humzah D Kerscher M. Cannula versus sharp needle for placement of soft tissue fillers: an observational cadaver study. Aesthet Surg J 2017;38:73–88. 27986754 10.1093/asj/sjw220

[16] Al-Hage J Galadari HI. The needle versus cannula debate in soft tissue augmentation. Dermatol Clin 2024;42:69–77. 37977686 10.1016/j.det.2023.06.010

[17] Pavicic T Frank K Erlbacher K Neuner R . Precision in dermal filling: a comparison between needle and cannula when using soft tissue fillers. J Drugs Dermatol 2017;16:866–72. 28915281

[18] Fagien S Bertucci V von Grote E Mashburn JH. Rheologic and physicochemical properties used to differentiate injectable hyaluronic acid filler products. Plast Reconstr Surg 2019;143:707e–720e. 10.1097/PRS.0000000000005429 PMC7597953 30921116

[19] Faivre J Gallet M Tremblais E Trévidic P . Advanced concepts in Rheology for the evaluation of hyaluronic acid-based soft tissue fillers. Dermatol Surg 2021;47:e159–e167. 33492870 10.1097/DSS.0000000000002916 PMC8078113

[20] Lin F Goodman GJ Magnusson M Callan P . Movement of the Syringe during filler Aspiration: an ultrasound study. Aesthet Surg J 2022;42:1109–16. 35348575 10.1093/asj/sjac032

[21] Schelke LW Cassuto D Velthuis P Wortsman X. Nomenclature proposal for the sonographic description and reporting of soft tissue fillers. J Cosmet Dermatol 2020;19:282–8. 31456355 10.1111/jocd.13127

[22] Song WC Choi HG Kim SH Kim SH . Topographic anatomy of the zygomatic arch and temporal fossa: a cadaveric study. J Plast Reconstr Aesthet Surg 2009;62:1375–8. 18948070 10.1016/j.bjps.2008.06.037

[23] Wong CH Mendelson B. Facial soft-tissue spaces and retaining ligaments of the midcheek: defining the premaxillary space. Plast Reconstr Surg 2013;132:49–56. 23508054 10.1097/PRS.0b013e3182910a57

[24] Andretto Amodeo C Casasco A Icaro Cornaglia A Kang R . The suborbicularis oculi fat (SOOF) and the fascial planes: has everything already been explained? JAMA Facial Plast Surg 2014;16:36–41. 23807472 10.1001/jamafacial.2013.53

[25] Schelke L Cotofana S Velthuis P. Two new phenomena associated with filler injection. Aesthet Surg J 2023;43:NP134–NP135. 36268611 10.1093/asj/sjac271 PMC9972513

[26] Schelke L Decates TS Cartier H CotofanaVelthuis SP. Investigating the anatomic location of soft tissue fillers in noninflammatory nodule formation: an ultrasound-imaging-based analysis. Dermatol Surg 2023;49:588–95. 36942950 10.1097/DSS.0000000000003756 PMC10227930

[27] Yang M Gu Y Wang D Li J . Liposuction of the zygomatic arch area: a novel concept to improve the midface Contour. Aesthet Plast Surg 2022;46:1689–97. 10.1007/s00266-021-02765-8 35059815

